# Extended Use of Central Veno-Arterial Extracorporeal Membrane Oxygenation in Lung Transplantation for Patients with Pulmonary Arterial Hypertension

**DOI:** 10.1093/ejcts/ezaf256

**Published:** 2025-08-04

**Authors:** Mitsuaki Kawashima, Naohiro Ijiri, Chihiro Konoeda, Gouji Toyokawa, Keita Nakao, Yue Cong, Haruaki Hino, Hideo Kurosawa, Koichi Kashiwa, Michiko Ushio, Gaku Kawamura, Masahiko Ando, Shogo Shimada, Minoru Ono, Masaaki Sato

**Affiliations:** Department of Thoracic Surgery, The University of Tokyo Hospital, Tokyo 113-8655, Japan; Department of Thoracic Surgery, The University of Tokyo Hospital, Tokyo 113-8655, Japan; Department of Thoracic Surgery, The University of Tokyo Hospital, Tokyo 113-8655, Japan; Organ Transplantation Center, The University of Tokyo Hospital, Tokyo 113-8655, Japan; Department of Thoracic Surgery, The University of Tokyo Hospital, Tokyo 113-8655, Japan; Department of Thoracic Surgery, The University of Tokyo Hospital, Tokyo 113-8655, Japan; Department of Thoracic Surgery, The University of Tokyo Hospital, Tokyo 113-8655, Japan; Department of Thoracic Surgery, The University of Tokyo Hospital, Tokyo 113-8655, Japan; Department of Clinical Engineering, The University of Tokyo Hospital, Tokyo 113-8655, Japan; Department of Clinical Engineering, The University of Tokyo Hospital, Tokyo 113-8655, Japan; Department of Anesthesiology and Pain Relief Center, The University of Tokyo Hospital, Tokyo 113-8655, Japan; Department of Anesthesiology and Pain Relief Center, The University of Tokyo Hospital, Tokyo 113-8655, Japan; Organ Transplantation Center, The University of Tokyo Hospital, Tokyo 113-8655, Japan; Department of Cardiovascular Surgery, The University of Tokyo Hospital, Tokyo 113-8655, Japan; Department of Cardiovascular Surgery, The University of Tokyo Hospital, Tokyo 113-8655, Japan; Organ Transplantation Center, The University of Tokyo Hospital, Tokyo 113-8655, Japan; Department of Cardiovascular Surgery, The University of Tokyo Hospital, Tokyo 113-8655, Japan; Department of Thoracic Surgery, The University of Tokyo Hospital, Tokyo 113-8655, Japan; Organ Transplantation Center, The University of Tokyo Hospital, Tokyo 113-8655, Japan

**Keywords:** lung transplantation, pulmonary arterial hypertension, veno-arterial extracorporeal membrane oxygenation

## Abstract

**Objectives:**

Lung transplantation (LTx) for patients with pulmonary arterial hypertension (PAH) is associated with high postoperative morbidity and mortality. Extracorporeal membrane oxygenation (ECMO) supports patients’ haemodynamics during and after LTx. However, the optimal ECMO strategy, especially for patients with PAH, is debated. Here, we report our unique strategy for patients with PAH, using postoperative central veno-arterial (VA)-ECMO combined with delayed chest closure.

**Methods:**

This was a retrospective single-centre study of consecutive bilateral lung transplantations for adult patients with PAH performed between 2021 and 2024. Patients’ characteristics, perioperative ECMO strategy, and postoperative outcomes were reviewed.

**Results:**

During the study period, 20 PAH patients (idiopathic or hereditary PAH [n = 17], PAH secondary to collagen disease [n = 1], pulmonary veno-occlusive disease [n = 1], and Eisenmenger syndrome [n = 1]) underwent cadaveric LTx. Intraoperative support comprised either central VA-ECMO (n = 17) or cardiopulmonary bypass (n = 3). In 17 patients, central VA-ECMO was maintained postoperatively with temporary skin closure. The reason for postoperative central VA-ECMO was anticipated post-LTx heart failure due to PAH and suboptimal cardiac function. The median duration of ECMO support was 4 days (interquartile range: 2-4). There were 9 (45.0%) haemothorax evacuations while patients were on postoperative central VA-ECMO. No patients experienced haemodynamic collapse after LTx. All patients survived during the observation period, resulting in a 100% survival rate at both 90 days and 1 year (95% confidence interval: 83.2%-100%).

**Conclusions:**

Extended postoperative central VA-ECMO and delayed chest closure were feasible for patients with PAH who underwent LTx. Meticulous haemostasis is mandatory, given the high chance of haemothorax evacuation.

## Graphical abstract

**Figure:**
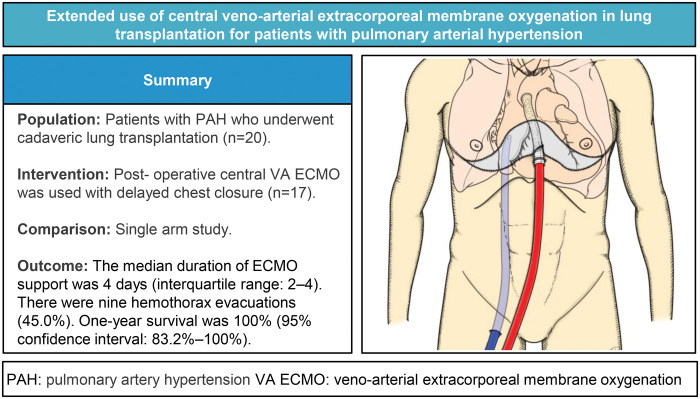

## INTRODUCTION

Lung transplantation (LTx) for patients with pulmonary arterial hypertension (PAH) historically has been associated with high postoperative morbidity and mortality.^1^ In PAH patients, this is largely driven by a higher probability of developing primary graft dysfunction because of impaired left ventricular function as well as overfilling from the hypertrophied right ventricle.^2–5^ Extracorporeal membrane oxygenation (ECMO) is a powerful tool to support patients’ haemodynamics during and after LTx. For patients with PAH, postoperative extended use of ECMO is reported as prophylactic or preventative veno-arterial (VA)-ECMO to mitigate the risk of primary graft dysfunction and heart failure.^3^^,^^6–12^ Postoperative VA-ECMO can be performed through peripheral^6^^,^^9–14^ or central cannulation.^7^^,^^13^ Our institution preferentially performs intra- and postoperative central VA-ECMO combined with delayed chest closure (DCC) for patients with PAH. Here, we report the results of our unique strategy, with the surgical details.

## METHODS

### Study design

All consecutive cases of cadaveric LTx for adult patients with PAH performed in our institution between January 2021 and December 2024 were retrospectively reviewed. Patients with PAH were enrolled in the study when their transplant surgery was performed. Regarding the exclusion criteria, patients who did not have a diagnosis of PAH were excluded. Additionally, cases of cadaveric single LTx, living donor LTx, and paediatric LTx were excluded. The study was approved by the Institutional Review Board of The University of Tokyo Hospital (2406-(9)). The need to obtain patient consent was waived because of the retrospective study design. Preoperative patient characteristics and postoperative outcomes were reviewed.

### Reporting guidelines

This retrospective observational study was performed and reported in accordance with the Strengthening the Reporting of Observational Studies in Epidemiology (STROBE) guidelines. A completed STROBE checklist is included as Supplementary File S1.

### Anaesthetic induction and transient peripheral VA-ECMO before establishing central VA-ECMO

In the operation room, an arterial line was placed in the radial artery under local anaesthesia before induction of general anaesthesia. After intubation, a central line and Swan-Ganz catheter were placed in the right internal jugular vein. With the patient in the supine position, the right inguinal area was incised, and peripheral VA-ECMO was established through the femoral vein (FV) and artery. In patients with an extremely high risk of cardiovascular collapse, peripheral VA-ECMO was established with local anaesthesia before anaesthesia induction. Given that most PAH patients in this study were young Asian women, the size of the arterial cannula was usually limited to 16- or 18-Fr, which is satisfactory for 70%-80% support of a patient’s cardiac output. For venous drainage, a 22- or 24-Fr cannula was used through the FV. This transient peripheral VA-ECMO is an important measure to stabilize a patient’s haemodynamics until the establishment of central VA-ECMO.

### Surgical details of intra- and postoperative central VA-ECMO with delayed skin closure

**Figure 1.**
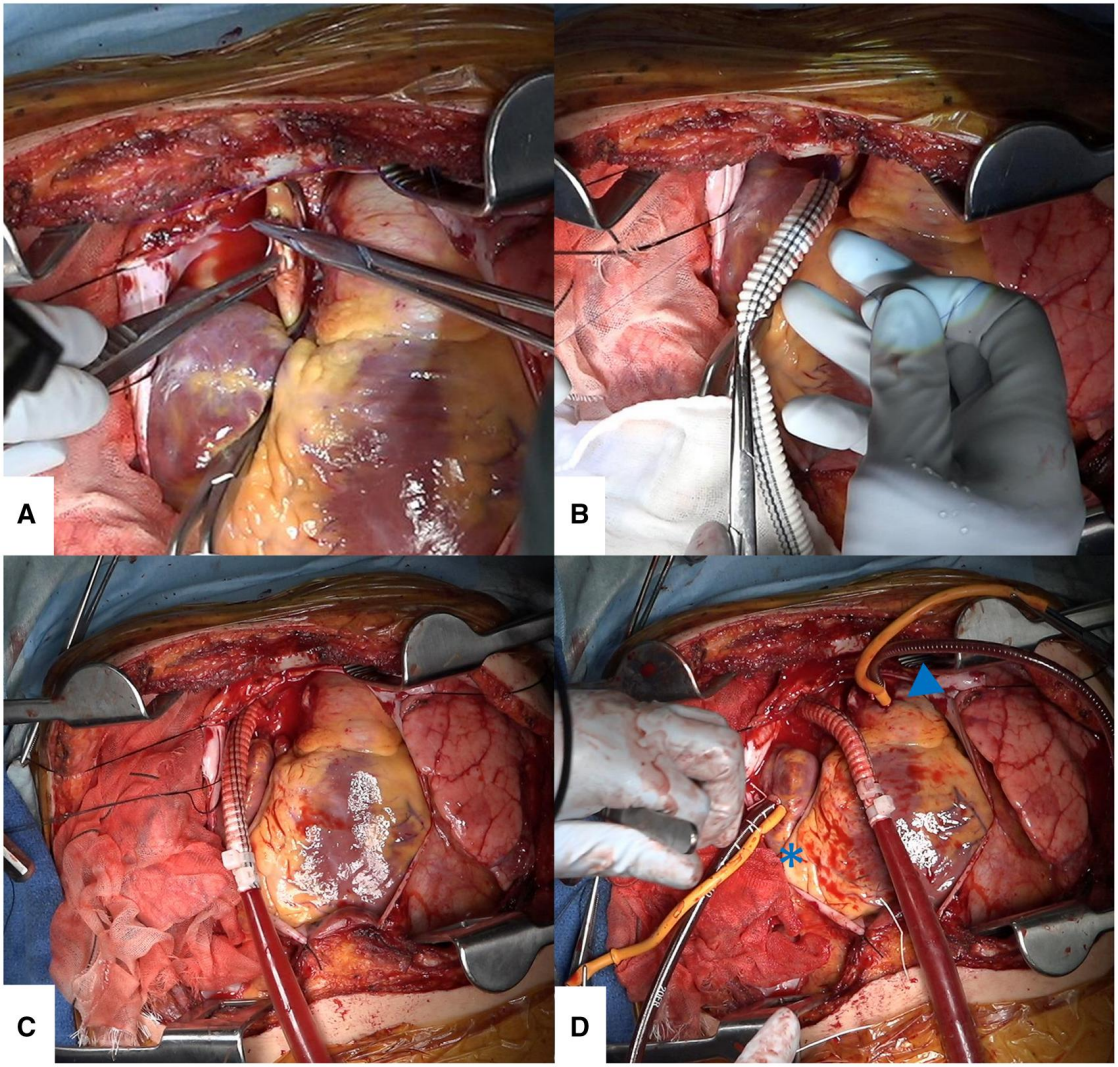
Intraoperative Findings: How to Establish Central VA-ECMO. (A) The ascending aorta is side-clamped and incised. (B) A woven aortic graft is sewn to the ascending aorta end-to-side. (C) A plastic catheter is inserted into the woven aortic graft and fixed. (D) A drainage cannula is inserted in the superior vena cava through the right atrium (asterisk), and a vent cannula is inserted into the pulmonary artery (arrowhead). ECMO, extracorporeal membrane oxygenation; VA, veno-arterial

While peripheral VA-ECMO was being established, a clamshell incision was made, and the chest was opened through the fourth intercostal space (**Video 1**). The pericardium was incised, and the caudal side of the sternal edge was covered with a pericardial flap using 2-0 silk suture to avoid mechanical trauma to the heart. After peripheral VA-ECMO was established, the ascending aorta was side-clamped and incised with an aortic punch. An 8-mm Gelsoft Plus (Terumo Co., Ltd, Tokyo, Japan) was sewn to the ascending aorta with 4-0 monofilament suture (**Figure 1A and B**). Fibrin glue was then applied to the aortic anastomosis site. An aortic perfusion cannula (Senko Medical Instrument Mfg. Co., Ltd, Tokyo, Japan) was inserted into the Gelsoft and secured with a band (**Figure 1C**). A purse string suture was placed in the superior vena cava (SVC) using 4-0 monofilament suture, and a 28-Fr drainage cannula was inserted (**Figure 1D**). The peripheral ECMO circuit was clamped and switched to the central cannulation setting for use with the cannulas mentioned above. A vent cannula was added to the PA main trunk to decompress a severely dilated PA (**Figure 1D**). Because of the three drainage cannulas (ie, SVC, FV, and PA), the drainage tube for the ECMO circuit must be branched twice to accommodate the cannulas. After restarting the ECMO circuit, the prostacyclin analogue infusion was discontinued. Stopping prostacyclin as early as possible is an important step because microvessels around the hilum are severely dilated due to prostacyclin in PAH patients, which makes hilar dissection challenging.

**Figure 2.**
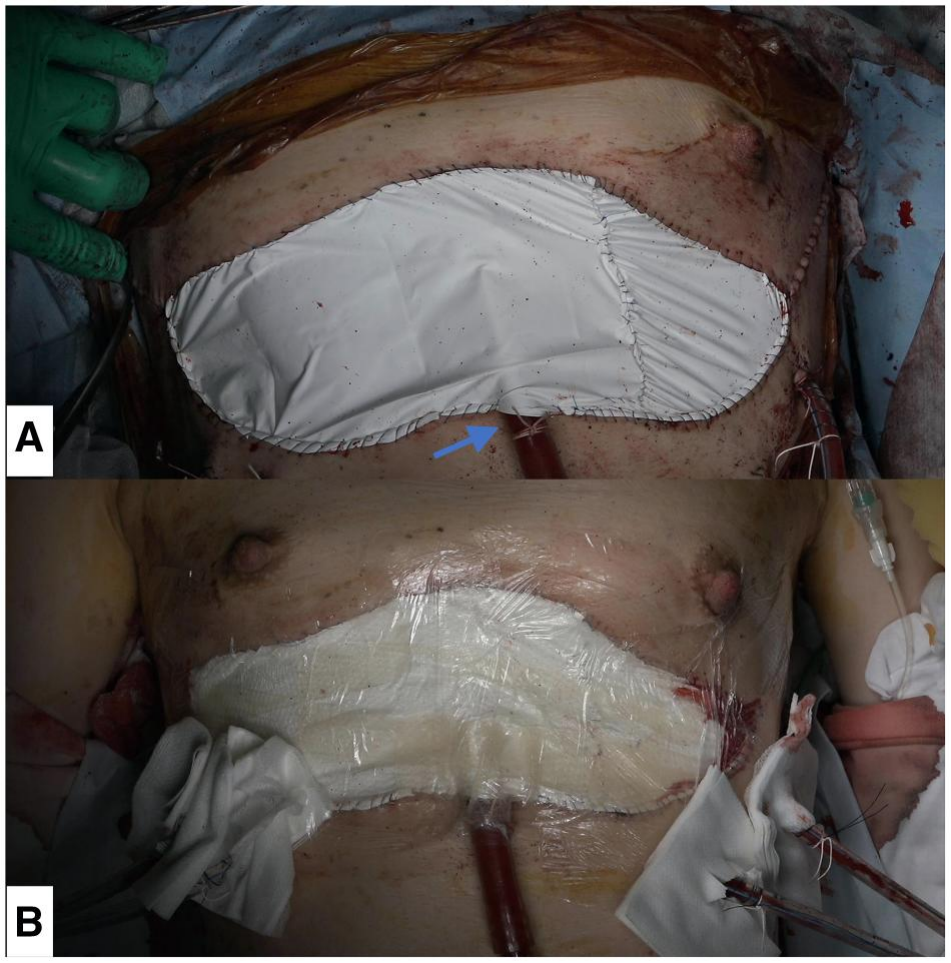
Postoperative Findings: Temporary Skin Closure. (A) Temporary skin closure using a Gore-Tex soft tissue patch with inlet space for the perfusion cannula. (B) Film dressing with a hydrocolloid wound dressing and polyurethane films

After implantation of the first lung, the PA vent was removed to allow some PA flow to the implanted lung. However, the PA vent was maintained until the implantation of the second lung if the mean PA pressure was sufficiently high to perfuse the implanted first lung. When the second lung implantation was complete, the SVC drainage cannula was removed, and the circuit was reconfigured to single drainage through the FV. Both arterial and venous cannulas were firmly secured to the skin with size 2 silk sutures. The chest remained open, and the skin was closed temporarily with a 0.1-mm thick, 15 cm × 20 cm GoreTex soft tissue patch (Japan Gore-Tex Inc., Tokyo, Japan) using 4-0 monofilament suture (**Figure 2A**). The wound was tightly covered with a hydrocolloid wound dressing (Karayahesive, 15 cm × 5 cm; Alcare Ltd, Tokyo, Japan) and a polyurethane-based wound dressing (Tegaderm Transparent Film Dressing, frame style, 10 cm × 12 cm; 3 M Japan Ltd, Tokyo, Japan) (**Figure 2B**).

Patients were moved to the intensive care unit with the central ECMO running. Over the next 2-3 days, ECMO flow was maintained and gradually weaned down. With Swan-Ganz monitoring, cardiac output was measured and used as an indicator of cardiac function recovery. Initially, after transplantation, patients’ left ventricles are not ready to receive the increased/normalized preload from the lungs, but as the patients recover, more blood flows through the heart and less blood flows through the central VA-ECMO. When cardiac output starts to exceed ECMO flow, and other haemodynamic parameters are stable, patients are ready for ECMO decannulation. For example, if a patient’s cardiac output is 1 l/min and ECMO flow is 2.5 l/min, the patient’s haemodynamics are ECMO-dependent, and the patient is not ready for ECMO decannulation. When a patient’s cardiac output is 2.0 l/min and ECMO flow is 1.5 l/min, more than half of the total blood volume flows through the patient’s heart, which suggests the recovery of the patient’s heart function.

At reoperation, the ECMO circuit was clamped, and if the patient’s haemodynamics remained stable, the ECMO cannulas were decannulated. The woven aortic graft was shortened and closed using 4-0 monofilament suture with proximal ligation using size 2 silk tie. The pericardium was reconstructed with a 0.1-mm thick, 12 cm × 12 cm GoreTex soft tissue patch (Japan Gore-Tex Inc.).

### Why use transient peripheral VA-ECMO first and central VA-ECMO with an aortic graft, thereafter?

To use central VA-ECMO postoperatively, the cannulas must be firmly secured to avoid inadvertent decannulation. Our method is to perform end-to-side anastomosis to the ascending aorta using a woven aortic graft instead of standard aortic cannula insertion with tourniquets. Our method provides good stabilization of the perfusion cannula, even postoperatively. However, a drawback is that side-clamping is required for aortic anastomosis, which increases afterload for the left ventricle and jeopardizes haemodynamics during anastomosis. Therefore, we establish peripheral VA-ECMO first to support the patient’s haemodynamics before establishing central VA-ECMO. This is actually a good way to prevent cardiovascular collapse even during anaesthetic induction. Because of size limitations of the femoral vessels, we found peripheral VA-ECMO unsatisfactory for the entire operation and prefer to use it only transiently. Additionally, postoperatively, maintaining central VA-ECMO has advantages over peripheral cannulations as the former provides more stable haemodynamic support and is associated with lower risks of differential hypoxia and limb ischaemia.

### Management of anticoagulation

Intraoperatively, 2000 units of unfractionated heparin was administered before cannulation, followed by 7-8 units/kg/hr of unfractionated heparin. After LTx, anticoagulation was temporarily withheld during patient transfer to the intensive care unit. If good haemostasis was achieved, heparin was restarted at 100-200 units/hr. If haemostasis was deemed unstable, anticoagulation was withheld or nafamostat mesylate 10-20 mg/hr was used as an anticoagulant.

To confirm adequate anticoagulation, activated clotting time (ACT) was measured 4 times daily at 6-hour intervals, and activated partial thromboplastin time (aPTT) was measured at least twice daily at 10-12-hour intervals. Both evaluations were performed more frequently when necessary. Other coagulation markers, such as thrombin-antithrombin complex and plasmin-alpha2-plasmin inhibitor complex were also monitored once daily, during ECMO.

The target ranges for anticoagulation were as follows: ACT, 160-200 seconds, and aPTT, 35-50 seconds. Considering the risk of bleeding, the lower half of these anticoagulation targets was preferred unless there were signs of thrombus formation in the ECMO circuit. Based on ACT, aPTT, thrombin-antithrombin complex, and plasmin-alpha2-plasmin inhibitor complex results, as well as the presence/absence of bleeding or a thrombus, anticoagulation was optimized by multidisciplinary discussion along perfusionists, intensivists, and thoracic surgeons.

### Management of haemostasis

Haemostasis behind the hilum (eg, bronchial arteries and lymphatic vessels) is a key for success with our method. The pulmonary ligament is another critical area. Oozing/bleeding at reoperation is sometimes identified in these areas. We use ligation, clipping, LigaSure Maryland, 23 cm (Medtronic Japan Ltd, Tokyo, Japan), or a combination of these methods, to achieve complete haemostasis. Fibrin glue, such as TachoSil (CSL Behring Japan, Ltd, Tokyo, Japan) can also be used. Patients with PAH usually have dilated microvessels due to the use of prostacyclin analogues. Therefore, haemostasis around the hilum is of utmost importance.

### Data analysis

A sample size calculation was not performed for this single-arm study. Continuous variables are presented as descriptive statistics with median and interquartile range because of the small sample size. No missing data were observed or handled in this study. The trajectories of ECMO flow and cardiac output were illustrated with GraphPad Prism version 10 for Windows (GraphPad Software, Boston, MA, USA). As no events occurred, Kaplan-Meier survival analysis could not generate a confidence interval. Instead, the 95% confidence interval for the observed 100% survival rate was calculated using the Clopper-Pearson method (EZR version 1.68 in R Commander; R Foundation for Statistical Computing, Vienna, Austria).

### Statistical approach and null hypothesis

Given the retrospective and single-arm study design, no formal statistical comparisons were performed. However, for the purpose of clarity and to align with scientific rigor, we state the following null hypothesis: “The extended use of central VA-ECMO in LTx is not associated with superior outcomes compared with those without ECMO or those with extended peripheral VA-ECMO for patients with PAH.”

## RESULTS

Twenty PAH patients (idiopathic or hereditary PAH [*n* = 17], PAH secondary to collagen disease [*n* = 1], pulmonary veno-occlusive disease [*n* = 1], and Eisenmenger syndrome [*n* = 1]) underwent cadaveric LTx (**Table 1**). The patients’ mean PA pressure was 59.5 ± 18.1 mmHg, which was 84% ± 24% of the systemic blood pressure at the time of LTx enrolment. All patients received two or three PAH-specific oral medications, and 18 patients were receiving continuous infusions of prostacyclin analogues (**Table 1**).

**Table 1.** Patients’ Characteristics

	(n = 20)
Age	29.5 [26.5, 40]
Male sex (%)	7 (35.0 %)
Group 1 pulmonary hypertension	
Idiopathic/hereditary	17 (85.0%)
Secondary to collagen disease	1 (5%)
Pulmonary veno occlusive disease	1 (5%)
Eisenmenger’s syndrome	1 (5%)
Mean PAP^a^	61 [48.75, 70]
Mean PAP/mean systemic BP^a^	0.75 [0.86, 1.00]
WHO functional class (I/II/III/IV)^a^	0/0/20(100%)/0
BNP (pg/ml)^a^	75.9 [31.2, 127.6]
6 min walk test (meters)^a^	390 [360, 462]
PAH oral medications	
Dual	13 (65%)
Triple	7 (35%)
Doses of prostacyclin analogue (ng/kg/min)	
Epoprostenol (n = 10)^a^	92.5 [54.5, 106]
Treprostinil (n = 8)^a^	85.5 [57.5, 100]

Continuous variables are expressed as median and quartiles because of the small sample size.

Abbreviations: BP, blood pressure; BNP, brain natriuretic peptide; PAH, pulmonary artery hypertension; PAP, pulmonary artery pressure; WHO, World Health Organization.

a Measured at the time of lung transplant enrollment.

Intraoperative support was via either central VA-ECMO (*n* = 17) or cardiopulmonary bypass (*n* = 3). Cardiopulmonary bypass was chosen when patients required concomitant cardiac repair or reconstructive surgery for the main PA trunk in the following situations: atrial septal defect (*n* = 1), patent ductus arteriosus (*n* = 1), and giant pulmonary artery aneurysm (*n* = 1). This decision between cardiopulmonary bypass and ECMO was made solely for cases of concomitant cardiac surgery because our standard mechanical support during LTx is ECMO, given the advantages of less anticoagulation and better haemostasis. In 17 patients, central VA-ECMO was maintained postoperatively with temporary skin closure (**Table 2**). The median duration of postoperative ECMO support was 3.3 ± 1.1 days (**Table 3**). There were 9 (45.0%) haemothorax evacuations while patients were on postoperative central VA-ECMO. Tracheostomy was performed for 14 (70.0%) patients, and the duration of ventilator support was 15.0 ± 8.1 days. The lengths of intensive care unit stay and hospital stay were 17.0 ± 5.3 days and 53.6 ± 27.0 days, respectively (**Table 3**). No patients experienced haemodynamic collapse after LTx. The duration (interquartile range) of the follow-up period was 452.5 (288.5-621) days. All patients survived during the observation period, resulting in a 100% survival rate at both 90 days and 1 year (95% confidence interval: 83.2%-100%).

**Table 2.** Modes of Circulatory Support during and after Lung Transplantation

	(n = 20)
Intraoperative cardiopulmonary bypass	3 (15.0%)
Intraoperative central VA ECMO	17 (85.0%)
Intraoperative peripheral VA ECMO	0 (0%)
	(n = 20)
Postoperative central VA ECMO	17 (85.0%)
Postoperative peripheral VA ECMO	0 (0%)

Abbreviations: ECMO, extracorporeal membrane oxygenation; VA, veno-arterial.

**Table 3.** Postoperative Outcomes

	(n = 20)
Duration of ECMO support (days)	4 [2, 4]
Duration of ventilator support (days)	14.5 [6.75, 21]
Tracheostomy	14 (70.0%)
Haemothorax	9 (45.0%)
Thrombosis	0 (0%)
Length of ICU stay (days)	16 [12.75, 19.25]
Length of hospital stay (days)	52.5 [38, 62.75]
90-Day survival	20 (100%)
1-Year survival	20 (100%)

Continuous variables are expressed as median and quartiles because of the small sample size.

Abbreviations: ECMO, extracorporeal membrane oxygenation; ICU, intensive care unit.

**Figure 3.**
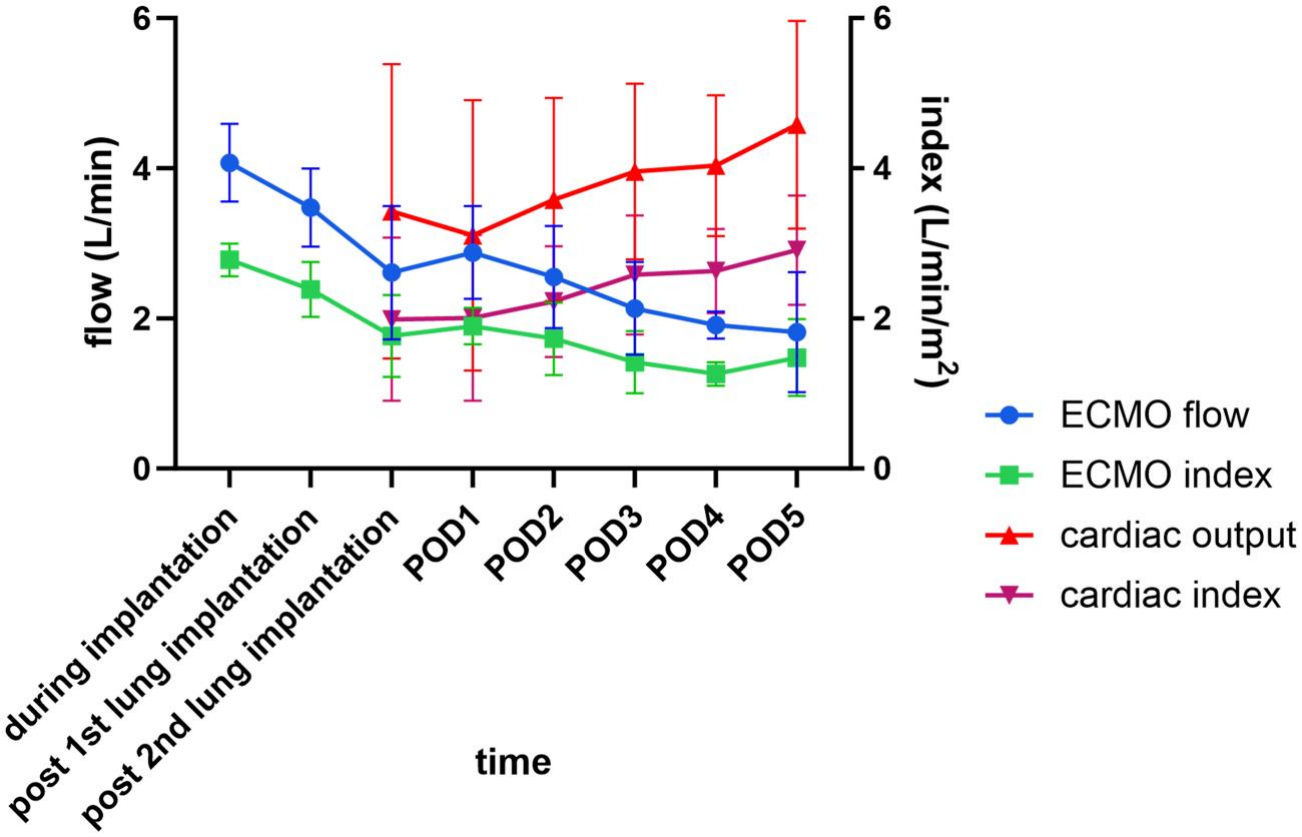
Intra- and Postoperative Trajectories of ECMO Flow, ECMO Index, Cardiac Output, and Cardiac Index. ECMO flow (L/min), ECMO index (L/min/m^2^), cardiac output (L/min), and cardiac index (L/min/m^2^) are shown as mean ± standard deviation. ECMO, extracorporeal membrane oxygenation; POD, postoperative day

The trajectories of ECMO flow, ECMO index, cardiac output, and cardiac index are summarized in **Figure 3**. During implantation, central ECMO with the triple drainage system achieved more than 120% of full flow (2.78 ± 0.22 l/min/m^2^). After the implantation of the second lung, the ECMO circuit was reconfigured to a single drainage system, with 80% of full flow (1.77 ± 0.54 l/min/m^2^). Immediately after transplantation, the patients’ cardiac output and ECMO flow were generally comparable, but on postoperative days 2-3, the patients’ cardiac output exceeded the support of their ECMO flow, suggesting recovery of left ventricular function and readiness for ECMO decannulation.

## DISCUSSION

LTx for patients with PAH requires special consideration regarding the surgery and postoperative care. These patients’ left ventricles are undertrained and can become overwhelmed with increased preload from the newly implanted lungs due to the sudden drop in pulmonary vascular resistance. The hypertrophied right ventricles pump excessive blood flow to the newly implanted lungs, which worsens ischaemia-reperfusion injury and increases pulmonary vascular resistance, which can cause strain on the right heart system.^2^^,^^3^ With these considerations, patients with PAH have high chances of left and right heart failure post-LTx. Prophylactic VA-ECMO is a useful measure to overcome these perioperative complexities. Hoetzenecker et al reported excellent results with 2-3 days of post-LTx peripheral VA-ECMO followed by slow weaning from the ventilator.^6^^,^^9^^,^^10^ Salman et al and Tudorache et al described a similar approach, but the authors extubated first and performed prolonged awake peripheral VA-ECMO for 1-2 weeks.^11^^,^^12^ Inoue et al reported prolongation of central VA-ECMO with DCC after 2-4 days.^7^ Glorion et al performed both peripheral and central approaches as postoperative support.^13^

In our early experience, we found that the peripheral cannulation strategy was unsatisfactory for our PAH patients for multiple reasons, as follows: (1) The risk of differential hypoxia hampers recovery of heart function. Preoperatively, a PAH patient’s heart suffers right heart failure and has a higher risk of postoperative cardiac collapse. (2) Typical Asian PAH patients are relatively short in stature and do not have sufficiently large diameter in the femoral arteries, which increases the risk of limb ischaemia. (3) Japanese PAH patients may have more advanced stages of PAH due to the long waiting times for LTx (ie, the average waiting time for LTx is 2.5-3 years in Japan because of donor shortage). These patients’ left ventricles are small because of the progressed PAH, and the ventricles cannot accommodate increased preload immediately after LTx. Therefore, we preferentially perform central VA-ECMO for PAH patients who undergo LTx in our institution, while peripheral cannulation is preferred for postoperative use in the American Association for Thoracic Surgery consensus document.^15^

This strategy of extended use of central VA-ECMO is particularly beneficial for patients with severe PAH, such as those with hypersystemic pulmonary artery pressure. There are no clear cutoffs regarding when to consider extended central VA-ECMO, and we usually rely on integrated decision-making on the basis of PAP, doses of prostacyclin analogues, and patients’ cardiac function. However, for example, if PAP is > 70% of systemic blood pressure (eg, arterial blood pressure: 120/70/90, PAP: 84/49/63), we would likely choose extended central VA-ECMO and not when PAP is <50% of systemic blood pressure (eg, arterial blood pressure: 120/70/90, PAP: 60/35/45). If unsure, we always place an aortic perfusion graft at the start of LTx and monitor the patient’s haemodynamics after bilateral implants. If a patient’s cardiac function recovers well, the aortic perfusion graft can be tied off for ECMO discontinuation at the end of surgery.

DCC is mandatory for postoperative central VA-ECMO. In our experience, DCC is highly appropriate in PAH patients. This approach allows patients to accommodate larger grafts and acquire a rich vascular bed, which is suitable to mitigate flow from the hypertrophied right ventricle. After 2-3 days of central VA-ECMO, these larger grafts become less oedematous and are a reasonable size for chest closure. To date, we have experienced satisfactory outcomes with this approach for PAH patients in our institution. However, a large prospective study is warranted, to determine the optimal perioperative ECMO strategy.

Vacuum-assisted closure is similar to DCC but is a salvage strategy for wound complications (ie, infection or dehiscence) after LTx.^16^ In our strategy, we do not combine vacuum-assisted closure with central VA-ECMO because of the risk of cannula compression and kinking, which may hamper ECMO flow.

The limitations of this study are the retrospective single-arm design, limited number of patients, and short follow-up period. Therefore, it was unfeasible to examine the null hypothesis in this study, and a large-scale future investigation is required. Notably, this study included the period of the coronavirus disease 2019 pandemic. However, the pandemic did not appear to markedly affect our study because our LTx volume was maintained during the pandemic. This is probably because our medical centre is small, and the number of transplants remained within the manageable range even during the pandemic.

## CONCLUSION

Extended postoperative central VA-ECMO combined with DCC is feasible for patients with PAH who undergo LTx. Meticulous haemostasis is mandatory, given the high chance of haemothorax evacuation.

## Supplementary Material

ezaf256_Supplementary_Data

## Data Availability

The data underlying this article are available in the article and in its online supplementary material.
